# Dissatisfaction with the laboratory services in conducting HIV related testing among public and private medical personnel in Tanzania

**DOI:** 10.1186/1472-6963-8-171

**Published:** 2008-08-11

**Authors:** SG Mfinanga, A Kahwa, G Kimaro, A Kilale, S Kivuyo, M Senkoro, B Ngowi, R Mtandu, B Mutayoba, E Ngadaya, K Mashoto

**Affiliations:** 1 Muhimbili Medical Research Centre, National Institute for Medical Research (NIMR), Dar es Salaam, Tanzania; 2 Centre for International Health (CIH), University of Bergen, Bergen, Norway; 3 Haydom Lutheran Hospital, Mbulu District, Manyara Region, Tanzania; 4 National Institute for Medical Research (NIMR), Headquarters, Dar es Salaam, Tanzania

## Abstract

**Background:**

A comprehensive care and treatment program requires a well functioning laboratory services. We assessed satisfaction of medical personnel to the laboratory services to guide process of quality improvement of the services.

**Methodology:**

A cross-sectional survey in 24 randomly selected health facilities in Mainland Tanzania was conducted to assess the satisfaction of the medical personnel with the laboratory services.

**Results:**

Of 235 medical personnel interviewed, 196 were valid for analysis and about one quarter were dissatisfied with the laboratory services. Personnel dissatisfied with the services were 38.3% in timely test result, 24.5% in correct and accurate results and 22.4% in clear complete results. The personnel in public laboratories were more dissatisfied with timely test results (OR = 3.6, 95% CI 1.8, 7.3), correct results (OR = 4.1, 95% CI 1.6, 10.8) and clear complete results (OR = 5.0 95% CI 1.6, 15.2). Personnel dissatisfied with the services in 15 laboratories sending specimens to referral laboratories, varied from 13% in availability of equipment to 57% in timely results feedback from the referral laboratories. Personnel dissatisfied with the services in 14 referral laboratories, varied from 28.6% in properly identified specimen to 42.9% in clear, accurate test request and communication.

**Conclusion:**

About one quarter of medical personnel in sending or receiving laboratories were dissatisfied with the services. Comparing the personnel in public and private, the personnel in public laboratories were 4 times more dissatisfied with the timely test and correct results; and 5 times more dissatisfied with clear and complete test results.

## Background

Tanzania is scaling up prevention, care and treatment of all communities affected with HIV/AIDS. The scaling up involves expanding the Voluntary Counseling and Testing (VCT), Prevention of Mother to Child Transmission (PMTCT) services, and increasing Antiretroviral (ART) availability and use [1]. Among other supporting systems, strengthening of laboratory services for enhanced diagnosis and emphasized continuing education for laboratory staff are of great importance.

As the delivery of Antiretroviral Therapy (ART) is largely dependant on diagnosing HIV infection and staging HIV/AIDS disease, there is a need to support the laboratory services for supporting HIV interventions. A good laboratory service is important to the medical personnel, making the diagnosis and staging the disease, as well as to the intended patients. Poor laboratory services may have a serious implication to the patients. Any laboratory should have a written policy focusing on customer's satisfaction, and should periodically measure and evaluate their customer's satisfaction [2,3]. In most cases, surveys for laboratory service are conducted, but authorities often fail to integrate the results into the continuous quality improvement and strategic planning processes. Moreover, most of laboratory management, do not often act upon customer service feedback [4].

Laboratory management staff should review the medical personnel feedback report and use it to improve the laboratory performance. The Quality in Laboratory Medicine (IQLM) forum proposed indicators for satisfaction with laboratories services. One of the key and often overlooked measures from the proposed list is clinician satisfaction, in which the IQLM called both a post-analytic and system indicator. Medical personnel satisfaction barely is addressed in CLIA. Laboratory quality improvement process requires laboratories to communicate certain specific information back to medical personnel who referred specimens to the laboratories [5]. In a recent study, of primary care physician practices (family practice), researchers found out that errors occurred throughout the spectrum of pre- and post analytic steps in the testing process in family physician's offices [6]. Evaluations of medical personnel satisfaction with laboratory services have not been done before in the country. Therefore, this survey assessed satisfaction of medical personnel to services provided by public and private laboratories in Tanzania, so as to guide process of quality improvement of the services for testing HIV infection and monitoring treatment.

## Methods

This was a cross-sectional baseline survey which was conducted from February to March 2007, in a total of 24 health facilities with laboratory services. These laboratories were randomly selected from a list of all public and privately owned laboratories in Tanzania mainland. Only facilities with laboratory services qualified to conduct HIV testing and provision of ARV were eligible for the survey.

The research tools were pre-tested at two government hospital laboratories named Temeke and Amana, and one private hospital named Hindumandal. The tools were then modified to suite the need of the survey. Three research teams, of three scientists each collected the data. Each team visited seven facilities with laboratory services.

Trained research assistants who were graduates from medical schools, administered the semi open ended questionnaires to the medical personnel. Nurses and physicians were interviewed regarding the satisfaction on laboratory services, while laboratory technicians interviewed concerning satisfaction with referral laboratory services, termed here as specimens sending and receiving laboratories. The team interviewed only in charge of laboratory services for sending and receiving laboratories. A total of 21 facilities were visited with an additional of 3 facilities that were involved in referral laboratory services only, making a total of 24 facilities.

The study plan was to interview 12 medical personnel per facility for the 21 facilities for client's satisfaction with laboratory services. These medical personnel were randomly selected from the facility lists. An additional one laboratory technician per facility was planed to be interviewed for satisfaction with referral laboratory services. Unfortunately some of selected medical personnel from the lists were not available on the day of interview and hence this caused variation of numbers of personnel interviewed per facility. Within the 21 facilities with laboratory services, only 12 laboratories had function of sending and receiving specimens (referral function) for further testing or quality assurance. In addition, interviews were conducted in additional three laboratories for satisfaction with referral function. Therefore in total, 14 facilities saved as both specimen sending and receiving function and one laboratory had no specimen receiving function and therefore making a total of 15 laboratories as shown in table 1.

**Table 1:** Facilities and number of personnel analyzed per facility

Medical personnel Interviewed for satisfaction with laboratory services		Specimen sending or receiving laboratories	
	
Facilities (Hospitals)	Frequency	Facilities (Hospitals)	Frequency
Agakhan	8	Agakhan	1
Bombo	11	Dodoma	1
Bugando	13	Ilembula	1
Huruma	12	Machame	1
Ilembula	6	Mafinga	1
Iringa Regional	11	Mikocheni	1
KCMC	15	Mount Meru	1
Machame	12	Mwananyamala	1
Mafinga	6	Sabasaba Health Centre	1
Mawenzi	10	Sekoutoure	1
Mbeya Referral	8	Selian	1
Mbeya Regional	8	Tosamaganga	1
Mikocheni	8	Tumbi	1
Mount Meru	8	Vywawa	1
Muhimbili	9	Morogoro	1
		
Mwananyamala	8	Total	15
		
Sekoutoure l	8		
Selian	12		
Tosamaganga	7		
Tumbi	8		
Vywawa	8		

Total	196		

In total, 235 medical personnel were interviewed, but after data cleaning a total of 196 personnel were valid for analysis as shown in table 1. A total of 235 out of expected 252 medical personnel others than laboratory technicians were interviewed, and 196 records of the personnel were valid for analysis of the satisfaction with laboratory services. A total of 15 laboratory technicians were interviewed. All interviewees that were available during the interview day, accepted to be interviewed, and this was referred as 100% response rate. The medical personnel were interviewed regarding the satisfaction on time, correctness, accuracy and completeness of the results.

More information about laboratory tests was obtained including, courteous communications, properly identified specimens, specimen containing pertinent clinical information, properly collected and transported specimens and timely feedback on their results. The satisfaction was measured using the dichotomy method and therefore the indifferent response was not allowed.

### Data analysis

The data collected was double entered, cleaned and coded using Epi-info version 6 (Centres for Disease Control and Prevention, Atlanta, GA, USA). Analysis was done using SPSS version 14 for Windows (SPSS Inc, Chicago, IL, USA). Gender and age grouping showed no significant association in all satisfaction indictor variables for laboratory services. Private and public laboratories were used as a comparison groups for various satisfaction indicator variables as shown in the result tables. Pearson Chi-squares were used to compare group differences for the categorical variables. Differences were considered statistically significant if p = 0.05. Stratification and logistic regression analysis were carried out to assess and adjust for interaction and confounding effect of education on regions and turnout in either public or private laboratory. Adjusted odds ratios with 95% confidence intervals are reported where appropriate.

### Ethical issues

Ethical clearance was obtained from National Institute for Medical Research Tanzania. Consent was sought from relevant administration of the hospital surveyed. Detailed information on the purpose of the survey and benefits were explicitly explained to each enrollee. The informed consent was requested from each of personnel who were involved in the study.

## Results

### Medical personnel dissatisfaction with laboratory services

Data from a total of 196 medical personnel with mean age of 39.7 (10.4) years from both public and private laboratory facilities were analyzed to determine whether they are satisfied or dissatisfied with the laboratory services. Respondents were from laboratory facilities in eight regions. There was no statistical significance difference in number of medical personnel analyzed between private and public laboratory facilities from all regions. Medical personnel from public laboratory were more dissatisfied with the laboratory procedures than their counterpart from private laboratories.

Table 2 shows that about three quarter of the medical personnel were satisfied with the laboratory services. However the proportion of the Medical personnel being dissatisfied were 38.3% in timely test result, 24.5% in correct and accurate results and 22.4% in clear complete results. There were differences in satisfaction with the laboratory services between the public and the private medical personnel analysed. Medical personnel working with public laboratories were more dissatisfied with timely test results (OR = 3.6, 95% CI 1.8, 7.3), correct results (OR = 4.1, 95% CI 1.6 – 10.8) and Clear Complete results (OR = 5.0 95% CI 1.6, 15.2).

**Table 2:** Proportions and Odd Ratios of medical personnel dissatisfied with laboratory services

Risk of dissatisfaction	Public n/N (%)	Private n/N (%)	All n/N (%)	OR (95% CI)
Timely test results	67/141(47.5)*	8/55(14.5)	75/196(38.3)	3.6 (1.8 – 7.3)
Correct results as per test requested	44/141(31.2)*	4/55(7.3)	48/196(24.5)	4.1 (1.6–10.8)
Accurate results	40/141(28.4)	8/55(16.7)	48/196(24.5)	1.9(0.9 – 3.7)
Clear Complete results	40/137(29.2)*	3/55(5.5)	43/192(22.4) **	5.0(1.6 – 15.2

*p = 0.001

** = does not add up to 196 owing due to missing value

Table 3 shows that the proportion of laboratory technicians dissatisfied with the laboratory services in 15 sending laboratories varied from minimum of 13% in availability of equipment to 57% in timely results feed back from receiving laboratory. The proportion of medical personnel dissatisfied with the laboratory services in 14 receiving laboratories varied from minimum of 28.6% in properly identified specimen to 42.9% in clear and accurate test request and communication between laboratories (table 4).

**Table 3:** The proportions of sending laboratories dissatisfied with specimen referral services

Risk of dissatisfaction	Public (%)	Private (%)	All (%)
Availability of equipment at referral laboratories	1/8(12.5)	1/7(14.3)	2/15(13.3)
Communication between receiving and referral laboratories	1/8 (12.5)	3/7(47.9)	4/15 (26.7)
Timely results feed back from receiving laboratories	4/7(57.1)	4/7 (57.1)	
Clear result report	2/7(28.6)	2/7(28.6)	4/14* (28.6)
Result generated from referral laboratories	1/8 (12.5)	1/7(14.3)	2/15(13.3)

* = does not add up to 15 owing due to missing value

**Table 4:** The proportions of receiving laboratories dissatisfied with specimen referral services

Risk of dissatisfaction	Public (%)	Private (%)	All (%)
Properly identified specimen	3/10(30.0)	1/4(25.0)	4/14(28.6)
Clear and accurate test request	4/10 (40.0)	2/4(50.0)	6/14(42.9)
Properly collected and transported specimen	3/10(30.0)	1/4(25.0)	4/14(28.6)
Availability of equipment	4/10 (40.0)	1/4(25.0)	5/14(35.7)
Communication between laboratories	4/10 (40.0)	2/4(50.0)	6/14(42.9)
Timely results feed back to the sending laboratory	1/10(10.0)	1/4(25.0)	2/14(14.5)

## Discussion

Just as in other service oriented sectors, medical personnel satisfaction with laboratory service is of utmost important as a feedback for quality of laboratory services. Satisfaction is one of the outcome measures for health care services and it serves as a useful quality improvement tool, required by most clinical laboratories.

Most current researchers are less interested in correlations between client's characteristics with service satisfaction. However, client's satisfaction is an important feedback to quality of any service delivery and it important tool for quality improvement cycle. In the country, just like any other developing countries, studies of this nature are very limited. Generally in our study we found that about three quarter of medical personnel were satisfied with laboratory services, this is comparable with the findings of the studies done in USA [6,7] The USA study revealed that nursing personnel were most satisfied with the accuracy of test results, phlebotomy courtesy toward patients and notification of abnormal results [6]. Yet in another study physicians were not satisfied with laboratory services [8].

Our study shows that medical personnel working with public laboratories were over 3 times more dissatisfied with the timely test results than those working with private laboratories. This finding corresponds to that obtained by [9]. This fact is a reflection of deteriorating quality of service in public services. It is, therefore, important to implement interventions at point of care and treatment by improving laboratory information system especially on turn around time so as to improve laboratory services particularly in public sector. Continuous monitoring of providers of laboratory services can improve medical personnel satisfaction as it has been shown in developed countries [1,5,9].

According to [1], missed test results are common in clinical practice and may compromise patient safety. However, our study did not assess extent of missed results. Our findings show that over 50% of the laboratories sending specimens to other laboratories were dissatisfied with the timely results feedback from referral services in both private and public laboratories. In contrast, laboratories receiving specimens from other laboratories were dissatisfied with clear and accurate test request as well as with the communication. Improvement in laboratory services between sending and receiving laboratories is essential for operational efficiency and patient care. Effective communication channels need to be established to achieve these goals.

We consider that inclusion of both public and private laboratories as strength of the study, since trend of utilization of private facilities for care and treatment of HIV/AIDS affected individuals has been increasing in recent years. However, we were not able to analyze our data for satisfaction of service by different cadre of nurses and physicians, and facility level. Dropping of the sample size of medical personnel from 252 to 196 due to either unavailability of respondents for interview or missing information is a limitation though it could have not caused a major impact to the findings obtained because about 80% of information was analysed.

## Conclusion

About one quarter of medical personnel in sending or receiving laboratories were dissatisfied with the services. Comparing the personnel in public and private, the personnel in public laboratories were 4 times more dissatisfied with the timely test and correct results; and 5 times more dissatisfied with clear and complete test results

## Competing interests

The authors declare that they have no competing interests.

## Authors' contributions

SGM: Provided contribution on the study design, performed statistical analysis, drafted the manuscript and responded to the reviewer's comments. He also gave final approval of the version to be published. AKa: Participated in the design of the study, coordinating, drafting the manuscript, and responding to the reviewer's comments. GK: Participated in the study design, data collection and revising the manuscript. AKi: Involved in drafting the manuscript and revising it critically. MS: Provided substantial contributions to conception and design, acquisition of data, analysis and interpretation of data. SK: Participated in data collection, analysis and final approval of the version to be published. BN: Participated in the study design and data collection. RM: Participated in data collection, drafting the manuscript and revising it critically for important intellectual content. BM: Have made substantial contributions to conception and design of the study, acquisition of data, analysis and interpretation. EN: Participated in the design of the study, coordinating, drafting the manuscript, and responding to the reviewer's comments. KM: Involved in analysis drafting the manuscript and revising it critically for important intellectual content

## Pre-publication history

The pre-publication history for this paper can be accessed here:


